# High-security closed devices are efficient and safe to protect human oocytes from potential risk of viral contamination during vitrification and storage especially in the COVID-19 pandemic

**DOI:** 10.1007/s10815-021-02062-y

**Published:** 2021-01-11

**Authors:** Eleonora Porcu, Maria Lucrezia Tranquillo, Leonardo Notarangelo, Patrizia Maria Ciotti, Nilla Calza, Silvia Zuffa, Lisa Mori, Elena Nardi, Maria Dirodi, Linda Cipriani, Francesca Sonia Labriola, Giuseppe Damiano

**Affiliations:** 1 Infertility and IVF Unit, University of Bologna, Sant’Orsola University Hospital, Bologna, Italy; 2 Department of Medical and Surgical Sciences, University of Bologna, Bologna, Italy

**Keywords:** High-security devices, Closed system vitrification, Oocyte vitrification, COVID-19, Pandemic

## Abstract

**Purpose:**

The main purpose and research question of the study are to compare the efficacy of high-security closed versus open devices for human oocytes’ vitrification.

**Methods:**

A prospective randomized study was conducted. A total of 737 patients attending the Infertility and IVF Unit at S.Orsola University Hospital (Italy) between October 2015 and April 2020 were randomly assigned to two groups. A total of 368 patients were assigned to group 1 (High-Security Vitrification™ - HSV) and 369 to group 2 (Cryotop® open system). Oocyte survival, fertilization, cleavage, pregnancy, implantation, and miscarriage rate were compared between the two groups.

**Results:**

No statistically significant differences were observed on survival rate (70.3% vs. 73.3%), fertilization rate (70.8% vs. 74.9%), cleavage rate (90.6% vs. 90.3%), pregnancy/transfer ratio (32.0% vs. 31.8%), implantation rate (19.7% vs. 19.9%), nor miscarriage rates (22.1% vs. 21.5%) between the two groups. Women’s mean age in group 1 (36.18 ± 3.92) and group 2 (35.88 ± 3.88) was not significantly different (*P* = .297). A total of 4029 oocytes were vitrified (1980 and 2049 in groups 1 and 2 respectively). A total of 2564 were warmed (1469 and 1095 in groups 1 and 2 respectively). A total of 1386 morphologically eligible oocytes were inseminated by intracytoplasmic sperm injection (792 and 594 respectively, *P* = .304).

**Conclusions:**

The present study shows that the replacement of the open vitrification system by a closed one has no impact on in vitro and in vivo survival, development, pregnancy and implantation rate. Furthermore, to ensure safety, especially during the current COVID-19 pandemic, the use of the closed device eliminates the potential samples’ contamination during vitrification and storage.

## Introduction

Oocyte storage is used to preserve fertility for medical or social reasons [1–5] and to avoid embryo cryopreservation due to ethical, legal, and moral reasons [6–8]. It has also become a valuable tool for egg donor banks [9–14].

The principle of oocytes and embryo vitrification is to fully eliminate ice formation in the medium that contains the sample, in phases of cooling, storage, and warming of the procedure [15]. It can be achieved either by increased cooling and warming rates, or increasing concentration of cryoprotectants. Generally, both approaches are used. Cooling rates may vary according to the applied method, from rapid cooling (around 200 °C/min) to ultrarapid (up to 20,000–100,000 °C/min) [16, 17]. Typically, warming is performed rapidly. High cooling and warming rates may help to avoid chilling injury [18].

The cryoprotectant takes part in lowering the freezing point and in reducing or preventing the formation of ice crystals in aqueous solutions. The addition of cryoprotectants into vitrification solutions results in a significant increase in the degree of cellular dehydration. During the warming procedure, the transfer of the oocytes from a solution containing a high concentration of cryoprotectant to an isotonic solution can also lead to a reverse osmotic shock or over-swelling that could be lethal [19]. Therefore, during oocytes and embryo vitrification, a delicate balance between multiple factors is necessary to succeed.

Very high cooling and warming rates, high cryoprotectant concentrations solutions, and a low volume containing the samples are crucial requirements for success human embryos and oocytes’ vitrification [20–22].

Over the years, various authors have described different vitrification protocols. They differ in the type of cryoprotectants used (ethylene glycol, DMSO, PROH and sucrose, Ficoll and trehalose, alone or combined) [21]. To date, the most commonly used protocol for both oocyte and embryo vitrification involves the combination of 15% DMSO, 15% EG, and 0.5 M sucrose in a minimum volume (≤ 1 μL) [23]. Moreover, the authors experimented with different equilibrium and dilution parameters and support methods for cooling, storage, and heating.

Devices can be divided into two main categories: “open” and “closed.” The volume surrounding the samples during vitrification is minimal and similar to both systems (< 1 μL). “Open” vitrification devices (such as unprotected Open Pulled Straw [24], Cryotech [25], Cryolock [26], Cryoleaf [27], Vitri-Inga [28]) allow direct contact of the oocytes with liquid nitrogen (LN_2_) and usually provide very high cooling (20,000 °C/min) rates. Conversely, closed devices (such as CryoTip [29], High-Security Vitrification straw [30], Cryopette [31], Rapid-i [32], VitriSafe [33], and MicroSecure [34]) are unable to reach this cooling rate, but they have the advantage that samples can be stored completely isolated from the external environment. For this reason, the increasing use of closed vitrification devices for oocytes’ cryopreservation must face some scepticism. Nevertheless, it has been reported that the cooling rate obtained with closed devices generates a comparable survival rate if the warming rate is sufficiently high [35]. Some studies suggest that adequate exposure to cryoprotectant agents, before closed vitrification, and high warming rate can compensate for the reduction in cooling rates caused by the thermal insulation of the sample in closed systems [36–38]. Furthermore, in the literature, there are reported vitrification systems that do not require high cooling/heating rates and small volumes to obtain vitrified embryos or oocytes (such as I.C.E. Vitrification, Innovative Cryo Enterprises LLC, Linden, NJ) [39–41]. Despite the successful use of “open” carriers for vitrification, direct contact between the sample and LN_2_ may not be desirable because of the potential risk of cross-contamination between specimens and inadvertent exposure to contaminants present in the tanks [42–44]. The use of “closed” vitrification carriers circumvents these risks. Some reports confirm the presence of microorganisms, bacteria, and viruses, which may cause infections in LN_2_ despite its temperature is − 196.5 °C [43, 45]. During long-term cryopreservation, ice sediment accumulates in storage dewars a risk of microbial contamination to stored samples. Ice accumulates in LN_2_ by two general processes: ice forming in the atmosphere above an open dewar falls into the vessel and ice forming on cold surfaces of the dewar or inventory system that enters LN_2_. These ice crystals aggregate and entrap other materials, such as bacteria and fungal spores. Theoretically, if the LN_2_ and its vapors were contaminated, stored samples could also become contaminated; thus, the LN_2_ itself can be considered a potential source of pathogens during cryopreservation and long-term storage. This problem has led to serious concerns about the use of open devices in vitrification [46–48].

The main purpose and research question of the study are to compare the efficacy of high-security closed versus open devices for human oocytes vitrification. In the case of comparable efficacy, closed systems might offer an additional advantage as the samples are not in direct contact with LN_2_ and thus avoids hypothetical contamination particularly dangerous especially during and after the COVID-19 pandemic.

## Materials and methods

The study was approved by the local ethical committee. All the patients attending the Infertility and IVF Unit, at Sant’Orsola University Hospital (Italy), were stimulated with recombinant follicle-stimulating hormone and gonadotropin-releasing hormone analogues. Monitoring was performed with estradiol serum levels’ determinations and pelvic ultrasounds to measure the diameter of the follicles. Ovulation was triggered by an injection of subcutaneous human chorionic gonadotropin (hCG) when the leading follicle reached 18–20 mm diameter and estradiol serum concentrations were around 200 pg/mL for each mature. Vitrification was performed in the occurrence of supernumerary oocytes, failure of semen production, and risk of ovarian hyperstimulation syndrome. For ethical reasons, the Bologna Infertility and IVF Unit cryopreserve female gametes instead of embryos. For this reason, just a few fresh or warmed oocytes are inseminated each time.

## Study design

This prospective randomized study was designed to compare the efficiency of vitrification in open versus closed devices. The primary end-point was the survival rate after warming.

francesca.labriola2@unibo.itA total of 1377 oocytes were needed in each arm assuming a survival rate of 65% for High-Security Vitrification™ and 75% for Cryotop® (*α* 0.05 and power of 80%), based on the average results previously obtained (data not shown).

A total of 860 patients were recruited considering a mean of 4 oocytes per patient and an average cancellation rate of 20% due to personal choices, lack of ovarian response, no retrieved oocytes, or no supernumerary oocytes.

**Fig. 1:**
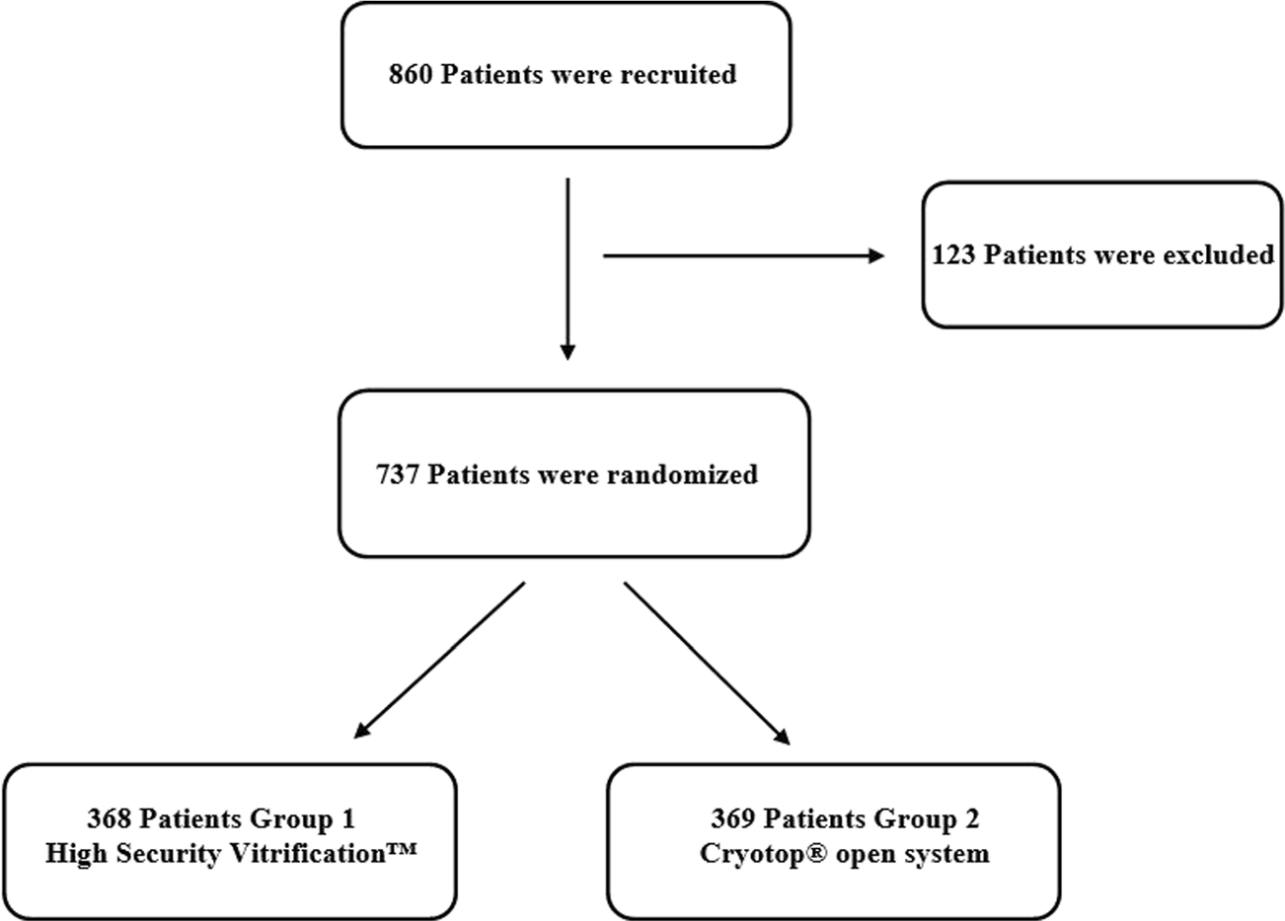
Flow chart of inclusion and randomization of patients

At the end of the randomization process, 737 patients were included in the study, with a final cancellation rate of 14.3% (123 patients). A total of 4029 MII oocytes were vitrified. Oocytes were randomly assigned to the closed group (group 1) or the open group (group 2) (Fig. 1).

### Oocyte vitrification method

Denuded oocytes were vitrified, by Vit Kit® (Irvine Scientific), using Kuwayama’s protocol [23]. Oocytes were placed in a pre-balanced 50 μL drop of HEPES solution (37 °C) that was immediately fused with 50 μL of Equilibration Solution (ES) for 3 min, followed by a second fusion with 50 μL of ES for 3 min. Then, the oocytes are transferred to a new 50-μL ES droplet for a variable time of 6–9 min and washed 5 times in vitrification solution (VS) before loading each oocyte on the chosen storage device. Finally, they were plunged in LN_2_. This procedure must be carried out in 1 min, the time necessary for permeating cryoprotectants to diffuse within the cytoplasm.

### Oocyte warming method

Thaw Kit® (Irvine Scientific) was used to warm oocytes according to Kuwayama’s protocol [23]. In the closed vitrification system, the upper extremity of the isolating protective straw was cut and the carrier was quickly submerged in 1 mL preheated thawing solution (TS) without direct contact with LN_2_. In the open system, the straw cap was initially removed under LN_2_ where the samples were in direct contact, and the carrier quickly submerged in 1 mL preheated TS. Regardless of the system used, the samples remained in the TS solution for 1 min at 37 °C. Then, oocytes were transferred into a drop of dilution solution (DS) at room temperature (25 °C) and incubated for 3 min. After two subsequent washing procedures in washing solution (WS) at room temperature for 6 min in total, oocytes were transferred into the fertilization medium (Vitrolife). Warmed oocytes were considered morphologically surviving if there were no dark/degenerated or contracted ooplasm and no cracked zona pellucida. Subsequently, oocytes were cultured at 37 °C (6% CO_2_ and 5% O_2_). Two hours post warming, oocytes were morphologically re-evaluated. Regardless of semen parameters, intracytoplasmic sperm injection (ICSI) was performed only in non-degenerate oocytes and completely rehydrated. The injected oocytes were cultured in individual 50-μL droplets of cleavage medium (Vitrolife) under oil (Vitrolife). The uninseminated oocytes surviving the warming process have been discarded because they are morphologically not eligible. Oocytes are considered morphologically not eligible if they have one or more of these anomalies: dark zona pellucida, large perivitelline space, dark or granular cytoplasm, cytoplasmic inclusions, vacuoles, and shape abnormalities.

### Fertilization and cleavage of warmed oocytes

Fertilization was assessed 16–20 h post ICSI by an inverted microscope (× 20).

Embryo quality was assessed on day 2 using Veeck’s classification [49].

### Embryo transfer

Embryos were transferred on day 2. Endometrial preparation was performed with patches containing estradiol hemihydrate: 300 μg/day for 11 days, and when the endometrial thickness reached 10 mm, 600 mg/day of micronized progesterone was added.

### Clinical outcomes

Pregnancy was assessed by serum hCG measurement 14–15 days after embryo transfer (value > 5 UI/L) and was then confirmed when at least one gestational sac was visualized at the transvaginal US after two further weeks. Miscarriage was considered the loss of a clinical pregnancy before the 20th week of gestation.

## Statistical analysis

Data were expressed as mean ± SD (standard deviation). Student’s *t* test was used to compare aged, frozen, and warmed oocytes. The chi-square test was used for oocyte’s survival, fertilization, cleavage, number of embryos transferred, pregnancy, implantation, and miscarriage rate. *P* values < .05 were considered statistically significant. All tests performed were validated through IBM SPSS Statistics (version 25.0).

## Results

Tables 1 and 2 show biological and clinical results obtained from vitrification-warming cycles with Hight Security Vitrification™ closed system (group 1) and Cryotop® open system (group 2).

**Table 1:** Comparison of the biological parameters of group 1 (HSV) and group 2 (Cryotop)

	Total	Group 1: HSV	Group 2: Cryotop	*P*
Patients	737	368	369	
Age (mean ± SD)	36.03 ± 3.90	36.18 ± 3.92	35.88 ± 3.88	.297
Age (range)	25–46	25–46	26–44	
Vitrification cycles	775	389	386	
Oocytes frozen (mean ± SD)	4029 (5.20 ± 3.45)	1980 (5.09 ± 3.09)	2049 (5.30 ± 3.80)	.055
Warming cycles	624	354	270	
Warmed oocytes (mean ± SD)	2564 (4.12 ± 1.53)	1469 (4.15 ± 1.65)	1095 (4.06 ± 1.41)	.147
Survived oocytes (%)	1835 (71.6)	1032 (70.3)	803 (73.3)	.096
Degenerated oocytes (%)	729 (28.4)	437 (29.7)	292 (26.7)	.096
Microinjected oocytes (mean ± SD)	1386 (2.23 ± 0.89)	792 (2.25 ± 0.95)	594 (2.20 ± 0.82)	.304
Normal fertilization (%)	1006 (72.6)	561 (70.8)	445 (74.9)	.104
Degenerated after ICSI (%)	89 (6.4)	54 (6.8)	35 (5.9)	.558
Cleaved embryos (%)	910 (90.5)	508 (90.6)	402 (90.3)	.994
Embryo grade I (%)	40 (4.4)	25 (4.9)	15 (3.8)	.480
Embryo grade II (%)	473 (52.0)	259 (51.0)	214 (53.2)	.543

**Table 2:** Number of embryos transferred and clinical outcomes

	Total	Group 1: HSV	Group 2: Cryotop	*P*
No. of transfer	545	297	248	
Transferred embryos (mean ± SD)	910 (1.47 ± 0.89)	508 (1.44 ± 0.92)	402 (1.49 ± 0.86)	.402
Pregnancy/transfer (%)	174/545 (32.0)	95/297 (32.0)	79/248 (31.8)	.953
Implantation rate (%)	180/910 (19.6)	100/508 (19.7)	80/402 (19.9)	.998
Miscarriage rate (%)	38/174 (21.8)	21/95 (22.1)	17/79 (21.5)	.927

A total of 737 patients for a total of 775 vitrification cycles were analyzed in the present study: 368 (389 vitrification cycles) and 369 (386 vitrification cycles) in groups 1 and 2 respectively.

Women’s age in group 1 (36.18 ± 3.92) and group 2 (35.88 ± 3.88) was not significantly different (*P* = .297). Women’s range age was 25–46 and 26–44 in groups 1 and 2.

A total of 4029 MII oocytes were vitrified. The closed vitrification system was used for 1980 oocytes, while 2049 were vitrified with the open system. There was no difference in the mean number of oocytes vitrified with the closed or the open system (5.09 ± 3.09 vs. 5.30 ± 3.80) (*P* = .055). A total of 624 warming cycles (*n* = 2564) were performed, 354 (*n* = 1469) and 270 (*n* = 1095) in groups 1 and 2 respectively. There was no difference in the mean number of warmed oocytes with the closed or the open system (4.15 ± 1.65 vs. 4.06 ± 1.41) (*P* = .147). Survival rate was 70.3% versus 73.3% (*P* = .096) in groups 1 and 2 respectively. A total of 1386 oocytes (2.23 ± 0.89) were microinjected, 792 (2.25 ± 0.95) and 594 (2.20 ± 0.82) in groups 1 and 2 respectively (*P* = .304). Fertilization rate (70.8% vs. 74.9%) and the cleavage rate (90.6% vs. 90.3%) showed no difference between the compared groups.

A total of 297 and 248 transfers were performed in group 1 and 2 respectively. The mean number of embryos transferred was similar in both groups (1.44 ± 0.92 vs. 1.49 ± 0.86) (*P* = .402). Moreover, there was no statistically significant difference between the closed and open groups in the pregnancy rate/transfer (32.0% vs. 31.8%), in the implantation rate (19.7% vs. 19.9%), or in the miscarriage rate (22.1% vs. 21.5%).

## Discussion

The development of a reliable and safe aseptic (closed) vitrification protocol is very important for human cell cryopreservation, especially after the Bielanski reports on the possibility of cross-contamination under LN_2_ that have raised concern about the use of open vitrification protocols [43–45]. So far, the only reported infection in LN_2_ occurred between blood samples stored in leaky plastic containers [50, 51]. In reproductive biology, however, no single report on disease transmission by LN_2_-mediated cross-contamination has been published. However, European Parliament’s guidelines (European Parliament and the Council of the European Union, 2004, 2006) have impelled scientists to look for solutions that would maintain vitrification in aseptic [52, 53]. The closed systems were examined and considered potentially harmful for the cells due to the lower cooling rates. At present, considering the increase in fertility preservation treatments, social egg freezing, and oocyte donation banking, storage can be expected to last up to several years. In such prolonged conditions, the main concern is to store biological material as safely as possible. A closed system device might guarantee the safest storage with the appropriate isolation from any detrimental factors [54, 55].

The policy of the University of Bologna Infertility and IVF Unit is to inseminate a limited number of oocytes and avoid the development of supernumerary embryos. Therefore, oocytes’ cryopreservation is offered to most patients.

The present study was able to show that there is no significant difference in survival rate between the devices: 70.3% in the closed HSV device versus 73.3% in the open Cryotop device. When compared to two other prospective randomized studies comparing open and closed devices [56, 57], this is the second report showing no difference in survival rate. Previously, only De Munck’s study [58] showed that there is no difference in survival rate: 93.7% in the closed HSV device versus 89.9% in the open CryotopSC device. Instead, the two previous reports found a significantly lower survival rate for the closed device: 57.9% versus 82.8% [56] and 82.9% versus 91.0% [57]. In the present study, the fertilization rate of the oocytes stored with close systems that survived warming is 70.8%. The result is comparable to the one obtained by De Munck (74.3%) and is in between the fertilization rates obtained by Paffoni (57.6%) and Papatheodorou (82.5%), whereas the fertilization rate of the open device is comparable to that obtained by Paffoni (73.0%) and Papatheodorou (73.4%) and is lower than that obtained by De Munck (81.4%). The degeneration rate after ICSI is not statistically significant between the two groups (6.8% in the closed HSV device vs. 5.9% in the open CryotopSC device). De Munck has observed a significantly higher degeneration rate after ICSI in the HSV arm (11.4% vs. 6.1%) and also Paffoni has observed a high degeneration rate with the closed device, 10.6% versus 6.3%, but it was not significantly different. In the present study, a clinical pregnancy rate of 31.8% in the open device and 32.0% in the closed device was obtained.

These results are in line with the study by Papatheodorou: 36.0% clinical pregnancy rate per cycle in the closed Vitrisafe device and 28.0% in the open Vitrisafe device, while Paffoni’s study reported a very low clinical pregnancy rate per cycle of 7.8% in the closed CryoTip device and 26.4% in the open CryoTop device. In the mentioned reports [57, 58], the average number of embryos transferred was very high (2.81 and 2.79 in Papatheodorou and 1.6 in De Munck). The present study shows a lower number of embryos transferred (1.47) compared to Papatheodorou’s and De Munck’s studies. It is difficult to compare our data with literature reports, as different types of open and closed devices are being used: closed CryoTip versus open CryoTop (Paffoni), closed versus open Vitrisafe device (Papatheodorou). In De Munck’s study only used the closed HSV versus open CryotopSC. Moreover, the study designs were different: the study by Paffoni was a retrospective inter-patient analysis, the study by Papatheodorou was a prospective randomized study where each recipient received oocytes from one type of device, while in the study by De Munck each recipient received oocytes from both devices.

Also, the studies by Papatheodorou and De Munck were conducted on sibling oocyte in a donation program.

On March 11, 2020, the World Health Organization (WHO) declared that the COVID-19 infection became pandemic due to its global geographical distribution [59]. Since then, several international scientific institutions in Reproductive Medicine, including the European Society of Human Reproduction and Embryology (ESHRE), American Society for Reproductive Medicine (ASRM), and Italian National Institute of Health (ISS), suggested suspending initiation of reproductive treatments, as well as fresh/frozen embryo transfers. However, the scientific community highlighted that fertility preservation due to oncological reasons or critical ovarian reserve should be performed storing gametes with the safest procedure [60–63]. Indeed, the Infertility and IVF Unity at the University of Bologna performed a number of oocyte storage for fertility preservation in cancer patients during the pandemic lockdown.

The results of the present study indicate that the high-security vitrification system is efficient and represents a valid strategy for storing biological samples in the safest way to reduce the potential risk of viral contamination. These results are of pivotal importance since many uncertainties remain around the effects of SARS-CoV-2 infection on ART**.**

## Data Availability

Not applicable
